# Effect of Finishing Protocols on the Surface Roughness and Fatigue Strength of a High-Translucent Zirconia

**DOI:** 10.1155/2023/8882878

**Published:** 2023-09-22

**Authors:** Larissa Araújo Lopes Barreto, Manassés Tercio Vieira Grangeiro, Pedro Henrique Condé Oliveira Prado, Marco Antonio Bottino, Amanda Maria de Oliveira Dal Piva, Nathalia de Carvalho Ramos, João Paulo Mendes Tribst, Lafayette Nogueira Junior

**Affiliations:** ^1^Department of Dental Materials and Prosthodontics, Institute of Science and Technology, São Paulo State University (Unesp), Eng. Francisco José Longo Avenue, 777, São José dos Campos, SP, Brazil; ^2^Department of Dental Materials Science, Academic Centre for Dentistry Amsterdam (ACTA), Universiteit van Amsterdam and Vrije Universiteit, Amsterdam, Netherlands; ^3^Department of Reconstructive Oral Care, Academic Centre for Dentistry Amsterdam (ACTA), Universiteit van Amsterdam and Vrije Universiteit, Amsterdam, Netherlands

## Abstract

**Purpose:**

In case of need for esthetical improvement of zirconia restorations, an individualization using extrinsic staining can be applied. This study aimed to evaluate the surface roughness and fatigue strength (survival) of high-translucency zirconia (3Y-TZP, YZ®HT, Vita Zanhfabrik) with extrinsic characterization and/or glaze.

**Methods:**

Sixty (60) zirconia discs (12 × 1.2 mm) were obtained, sintered, and randomly distributed among three groups (*n* = 20) according to the surface finishing protocol: C (control), C + G (extrinsic characterization followed by a glaze layer), and G (glaze layer). The surface roughness (Ra) was analyzed with a contact profilometer. Subsequently, the specimens were subjected to a fatigue load profile starting at 120 N during 20,000 cycles at 4 Hz frequency, with a 5% increase at each step until failure. The failed specimens were evaluated under a stereomicroscope. Surface roughness analysis was evaluated by using one-way ANOVA and post hoc Tukey tests (95%); while fatigue survival probability was analyzed with Kaplan–Meier and Mantel–Cox (log- rank, 95%).

**Results:**

One-way ANOVA revealed that surface roughness was affected by the finishing protocol, where C + G showed the highest mean value (0.46 ± 0.18 *µ*m)^A^ followed by G (0.30 ± 0.10 *µ*m)^B^, and C (0.19 ± 0.02 *µ*m)^C^. While for fatigue strength, the G protocol presented a higher mean value (243.00, and 222.36–263.63)^A^, followed by C + G (192.75 and 186.61–198.88)^B^ and C (172.50 and 159.43–185.56)^C^.

**Conclusion:**

Surface finishing protocols modify the surface roughness and fatigue strength of high-translucent zirconia. Regardless of the surface roughness, both glazing protocols improved the ceramic fatigue strength, favoring the restoration's long-term survival.

## 1. Introduction

Zirconium dioxide (ZrO_2_) is widely utilized in the field of health sciences, including dentistry, due to its remarkable mechanical properties [1]. However, the initial generations of zirconia were characterized by high strength at the expense of compromised esthetic characteristics [2]. In order to address the esthetic limitations, ceramic veneering was introduced as a means to enhance the appearance of the restorations while maintaining their mechanical performance. Nevertheless, the addition of the esthetic ceramic layer also brought about functional failures such as chipping and delamination [3]. With the development of newer generations of zirconia, featuring improved translucency, it became possible to utilize this ceramic material in a monolithic form, eliminating the risks associated with veneering ceramic and further enhancing the esthetic outcomes.

Despite the improved esthetic characteristics of zirconia, it may not always meet the high-esthetic demands of patients without additional processing. Therefore, for many restorations, an external shade characterization or staining procedure are necessary [4, 5]. This involves applying stains to the external surfaces of the restoration at the final stage of manufacturing, followed by the two firing procedures: one for staining and another for applying the glaze layer [4, 5]. Some studies have reported benefits for polished or glazed surfaces of uncharacterized zirconia [6]. However, there is no consensus among these studies regarding surface roughness, which may vary depending on the type of glaze material used [6, 7]. Furthermore, the advantages of using a glaze layer instead of external characterization have not yet been thoroughly investigated.

Recent studies highlight wear in glaze and stain layers of ceramics due to factors like antagonist contact and brushing [4, 5]. Limited data exist on zirconia's wear during use and the glaze's role in reliability [8–10]. Comprehensive characterization aids clinicians in balancing the esthetics and durability of zirconia restorations, including surface quality. Profilometry is an essential method for quantifying surface irregularities, and involves using stylus or noncontact probes to trace the surface. It provides valuable data on the surface topography, aiding in characterizing roughness features such as Ra, Rz, Rq, and Rmax. The selection of this method depends on the surface type and desired resolution, ensuring accurate and reproducible results for analysis and comparison [11, 12].

In vitro fatigue tests, which involve applying cyclic loads to simulate the clinical conditions experienced by the restorative materials in the oral cavity, have been widely utilized [13, 14]. These tests provide valuable insights into the probability of survival or failure of the ceramic restorations [15]. Therefore, they can also be employed to investigate the behavior of the stained zirconia. The objective of this study was to examine the impact of different finishing protocols (polishing, extrinsic characterization followed by glaze, and glaze) on the surface roughness, fatigue strength, and survival probability of a high-translucent zirconia material. The hypotheses formulated for this study were that the finishing protocol would not have a significant effect on (1) surface roughness and (2) fatigue behavior of the high-translucent zirconia. By testing these hypotheses, we aim to gain a deeper understanding of how different finishing protocols may influence the performance of stained zirconia restorations.

## 2. Materials and Methods

### 2.1. Specimens' Preparation

High-translucent zirconia blocks (Vita YZ® HT, Vita Zhanfabrik Bad Säckingen, Germany), were machined into a cylindrical shape (14 mm diameter) and then cut into discs (*N* = 60) on a precision cutting machine (Isomet® 1000, Precision Sectioning Saw, Buehler, Lake Bluff, Illinois, USA) under constant water cooling. Polishing (EcoMet™™/AutoMet™, Buehler, Illinois, USA) was performed with silicon carbide sandpapers with decreasing granulation (#400, #600, #800, and #1200). After cleaning in an isopropyl alcohol ultrasonic bath for 5 min, the samples were sintered (InFire HTC speed, Sirona Dentsply, France) to obtain final dimensions of up to final dimensions of 12 mm in diameter and 1.2 mm in thickness [16].

The calculation of the sample size was performed using the online calculator OpenEpi (http://www.openepi.com). The sample size was determined based on a two-sided comparison of means, with a significance level (*α*) of 0.05 and a power (1−*β*) of 0.80. The formula used for the calculation was based on the standard deviation (*σ*) observed in a pilot study and the desired minimum detectable difference (*δ*) between the groups. Considering these parameters, a total of 12 samples per group were determined to be necessary to achieve adequate statistical power for the study. However, 20 samples per group have been prepared and considered in this investigation.

Discs were then randomly assigned into three groups (*n* = 20) according to the finishing protocol: C, control, *G*, glazed, and C + G, characterized and glazed. In the C group, the discs did not receive any finishing protocol after sintering. Group G received a thin layer of glaze (VITA AKZENT Plus GLAZE POWDER, VITA Zahnfabrik, Germany) in one surface of the disc with a 1 : 2 powder/liquid dilute ratio (VITA AKZENT Plus POWDER FLUID, VITA Zahnfabrik, Germany) [17]. The glaze was applied with the aid of a brush to evenly distribute the product over the entire surface of the disc. After, glaze firing was carried out in the Vita Vacumat 6000MP oven (Vita Zahnfabrik, Germany). C + G group had one of the surfaces of the discs with an application of a thin layer of extrinsic characterization with a 1 : 2 powder/liquid dilute ratio (VITA AKZENT plus EFFECT STAINS, Vita Zahnfabrik, Germany), homogenized and applied using a brush, and were then fired in the Vita Vacumat 6000MP oven (Vita Zahnfabrik, Germany). After the stains had been fixed, a thin layer of glaze was applied following the same procedure of G group. All firing protocols followed the manufacturer's recommendations. The operator who manufactured the specimen and the one who performed data collection were, both, experienced dental professional with expertise in the specific procedures involved. Their qualifications and training were verified and met the required standards to ensure consistency and accuracy throughout the study. Given the nature of this in vitro study and the visible modifications to the specimen's surface, blinding the operator to the groups allocations were not feasible.

### 2.2. Surface Roughness

Surface analysis of the C (control), G (glaze), and C + G (extrinsic characterization followed by glaze) discs was conducted using a contact roughness meter (Surftest SJ 400, Mitutoyo, Tokyo, Japan). Three readings were taken at various angles on each specimen, and the average roughness parameter (Ra) was evaluated according to ISO 4287-1997 standards, utilizing a Gaussian filter and a 0.8 mm cutoff wavelength [18]. By calculating the average of the obtained values, an overall average roughness was determined for each group.

### 2.3. Fatigue Strength

To define the fatigue test loading profile, three specimens from each group were tested under the monotonic biaxial flexural strength test. For that, the discs were positioned on a circular metal base with 3.2-mm diameter balls equidistant from each other, forming a plane (ISO 6872:2015) [19], with the treated surface facing the tensile side. A 1.6-mm diameter indenter was attached to a universal testing machine (Emic DL-1000, Emic, São José dos Pinhais, PR, Brazil) and a 1,000 kgf load cell was used. The specimens were tested until failure.

Fatigue strength analysis was performed using the stepwise test [20]. From the mean load, the fatigue profile used in the stepwise test was determined. For that, the final load was calculated to be 360 N, and each step 15 N until the maximum load was achieved.

For this, the specimens were tested (in water, 4 Hz) using a mechanical fatigue machine (Biocycle, Biopdi, São Carlos, Brazil), with the same device configuration and disc position as used in the monotonic test [16]. A hundred Newton load for 5,000 cycles was applied for the indenter adaptation. Then, the fatigue loading profile started at 120 N with 15 N increase load step at each 20,000 cycles, until the specimens' failure. The use survival probability for a mission of 100,000 cycles at 150, 225, and 300 MPa were calculated. The failed specimens were analyzed using a stereomicroscope (Discovery V20, Zeiss, Jena, Germany) to analyze the features of the fracture and identify the origin of the failure.

### 2.4. Data Analysis

After confirming the assumptions of normality using the Kolmogorov–Smirnov test (95%), roughness data were tabulated for descriptive statistics for each group and then subjected to one-way ANOVA, followed by post hoc Tukey test, both with *α* = 0.05. The number of cycles and the load to fail during the fatigue test were analyzed by the reliability software using the Kaplan Meier and Mentel–Cox (log rank) (*p* < 0.05) survival analysis function. All analyses were performed using SPSS statistics software program (IBM, Chicago II, USA). The images obtained were qualitatively evaluated.

## 3. Results

### 3.1. Surface Roughness

One-way ANOVA revealed that the surface roughness of the groups was affected by the surface finishing protocol (*p* < 0.001). C + G showed the highest mean Ra value (0.46 ± 0.18 *µ*m)^A^ followed by G (0.30 ± 0.10 *µ*m)^B^, and C (0.19 ± 0.02 *µ*m)^C^.

### 3.2. Fatigue Strength

The means and 95% confidence interval (CI) of the strength of flexural fatigue and the cycles of fracture are presented in Table 1. By means of Kaplan Meier and log rank (Mantel–Cox) (*α* = 0.05) tests, specimens that were only glazed showed the highest fatigue strength and the number of cycles required to fracture; while polished (C) specimens showed the lowest mean fatigue strength, and the lowest number of cycles until fracture.

**Figure 1:**
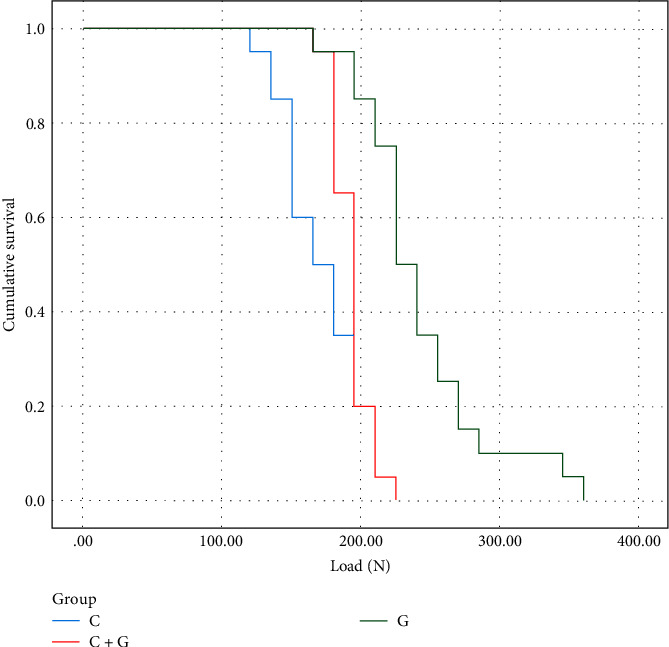
Survival graph of groups as a function of time (cycles) and load (N).

The survival graphs of the groups as a function of time (number of cycles) and load are presented in Figure 1. G protocol presented higher load and number of cycles to fail, followed by C + G and then C.  Table 2 shows the survival probability of each group through the loading steps and number of cycles. The 150 N load resulted in the survival probability of 40% lower for C compared to C + G and G. An increase in load to 225 N resulted in a survival probability of 0% for C and C + G; while G presented 50%. For 300 N, G survival probability decreased to 10%.

**Figure 2:**
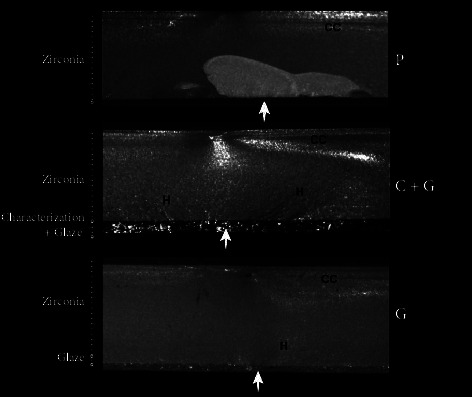
Stereomicroscope images of representative specimens from each group: P, polished surface of control group (C); C + G, characterized and glazed; G, glazed; CC, compression curl; and H, hackles. The white arrows indicate the locations of possible failure origins on the surface subjected to tensile stresses.

The fractographic analysis pointed out defects that possibly originated the fracture in the tensile side of the specimens (Figure 2, white arrows). The defects seem to be related to polishing surface procedures, or intrinsic porous in the characterization and/or glaze layer. Fracture features such as compression curl and hackles were observed and helped to locate the failures origin.

## 4. Discussion

This study aimed to investigate the effect of finishing protocols (C, C + G, or G) on a high-translucent zirconia surface roughness, fatigue strength, and survival probability. Even with translucency improvement of zirconia, it still presents opacity and a high value as characteristics. Clinicians should strive to offer restorative solutions that exhibit the lowest possible roughness and the highest achievable gloss, regardless of whether it involves a direct restoration or an indirect restoration [21–24]. Therefore, a characterization layer on zirconia can provide an appearance that is closer to the natural teeth.

Several studies have indicated that the application of a characterization layer can significantly increase surface roughness, leading to the accumulation of bacterial biofilm [25], which in turn may contribute to the periodontal inflammation [26]. In the current study, the roughness results revealed that the G or C + G protocols increased the surface roughness of the zirconia, contradicting the first hypothesis. Moreover, the C + G protocol exhibited higher average surface roughness compared to the other treatments, which is consistent with the findings reported by Bittar et al. [27], who also observed higher roughness values for translucent zirconia when staining and glaze were applied compared to the control group and glaze-only application. Conversely, another study by Manziuc et al. [28] reported a decrease in surface roughness after glazing, without the inclusion of a staining step. These discrepancies among the studies can be attributed to the methodological variations in roughness measurement techniques, differences in glaze materials utilized, and variations in the zirconia microstructure.

In a previous study by Jones et al. [29], participants who had restorations placed on their teeth were asked to evaluate the roughness of the restorations. The perception of roughness varied depending on the location of the restoration, with participants being more sensitive to changes in roughness on the lingual surfaces [29]. The authors of that study suggested that the maximum roughness of a finished restoration surface should not exceed 0.50 *μ*m in order to be undetectable by the patient. In the present study, the C + G group exhibited the highest mean roughness value (0.46 *µ*m), followed by the G group (0.30 *µ*m) and the C group (0.19 *µ*m). Although there were statistically significant differences between the groups, it is important to note that all groups fell within the clinically acceptable range for the surface roughness. Therefore, based on the maximum roughness threshold recommended by the previous research of Jones et al. [29], the finishing protocols applied in our study would be considered clinically acceptable in terms of surface roughness, even though some differences were observed between the groups.

By choosing materials with lower surface roughness, the dentist and the dental technician can reduce the risk of bacterial plaque retention and improve the long-term success of the dental treatments [10, 13, 21, 30]. Additionally, it can facilitate proper maintenance and cleaning of dental materials to prevent the buildup of bacterial plaque [25, 30]. According to the literature, the concept of “threshold surface roughness,” is the level of roughness at which bacterial plaque can adhere to a surface of a restorative material and was usually defined as 0.2 *µ*m [30]. The results of the present study are in agreement with the summarized data from the previous study when considering different dental ceramics submitted to the polishing or glazing [30]. In this sense, considering only the biofilm formation, the polishing protocol would be a more suitable finishing method than glazing for high-translucent zirconia [18].

It is worth to mention that zirconia with a characterization layer and glaze firing would be indicated for a better replica of the appearance and the optical properties of natural teeth. However, the final shade and translucency of ceramic restorations can be significantly influenced by the type of material, cement, and substrate [31]. The present study complements this information showing that the fatigue strength would not be affected by this protocol. This findings corroborate with the properties of zirconia crowns making them an ideal choice for the dental restorations that demand superior strength, precision, and durability [32].

A previous in vitro study by Zucuni et al. [33] compared the effects of two glaze application methods (brush and spray) on the fatigue strength and surface characteristics (topography and roughness) of high-translucency zirconia. The results indicate that both glaze application methods have the potential to create a smoother surface topography, but the glaze spray method produced thinner layers of material, which limited its ability to reduce the roughness when compared to the brush method. The present study corroborate with them, showing that both G and C + G are suitable methods for finishing the zirconia restorations.

In this study, the glaze layer was applied by the layering technique; however, spray or paste glaze can also be used as previously mentioned. Zirconia treated with spray glaze is suggested to have a rougher surface compared to the control one, presenting isolated “islands” of glaze on its surface [18, 34]. While, other investigation using the same zirconia and staining kit found half of the Ra values (0.2 *µ*m), suggesting that the evaluated zirconia after characterization followed by a paste glaze layer falls within the desired roughness threshold [4].

Despite the roughness assessment, the effect of a glaze layer on the fatigue performance of stained high-translucency zirconia is still scarce [34]. For that, a stepwise fatigue test was performed to predict the behavior of the material [35]. The results show that the G was the group with the highest fatigue strength and longest number of cycles to fail, rejecting the second hypothesis that the finishing protocol would not affect the fatigue behavior of a high-translucent zirconia. This may be related to the cooling cycle of the glaze firing, in which a layer of compressive stresses is generated in the zirconia, which increases the material mechanical strength [27]; or due to the fact that the glaze could fill the surface defects, increasing the ceramic structural reliability.

It is important to mention that the results of fatigue cycling tests can assist dental professionals in selecting the most suitable restorative material for a particular patient and evaluating the long-term performance of dental restorations. While fatigue cycling can provide valuable information about the mechanical properties of Yttria-stabilized zirconia high translucent and other materials, it is only one aspect of the overall evaluation of a dental restoration. Other factors such as esthetics, biocompatibility, and handling must also be considered when selecting the restorative material for each case [1–9, 36]. All ceramic materials have surface defects, which may be inherent to the fabrication of the CAD/CAM blocks or may have been generated during processing, such as cutting (or milling) and polishing. This observation provides justification for the control group having the lowest fatigue strength. During the polishing procedure, it is plausible that grains were dislodged from the surface [9, 25], leading to the generation of defects that can undermine the material's strength behavior as observed when conducted polishing after sintering [9], and before sintering [25]. Additionally, polishing procedures have been documented to induce the formation of a metastable rhombohedral phase, which might contribute to the increased degradation over time [26, 31].

This study focused on three missions to evaluate the zirconia performance in different load situations. For an anterior area, represented by 150 N [37], both C + G and G showed a superior behavior than C, suggesting that the glaze layer significantly improved fatigue survival. For 225 N, representing an intermediate load, only G had a survival probability of 50%, which dropped to 10% when evaluating the 300 N mission, corresponding to the posterior area [37, 38]. This result suggests that for intermediate and posterior loads, the presence of a stain layer decreased the fatigue survival compared to G.

One potential concern with C + G restorations is their resistance to wear, particularly in the case of stained zirconia restorations. When zirconia restorations are stained, a layer of colored glass is added to the surface of the material [38, 39]. This layer of stain is more susceptible to wear than the underlying zirconia, particularly if the stain is not properly attached to the zirconia surface. To evaluate the durability of zirconia restorations stained against wear, previous studies found that while staining can potentially be worn off the zirconia surface, the level of wear is still within acceptable limits of clinical use [4, 5]. In clinical practice, the durability of stained zirconia restorations against wear will depend on various factors, including the quality of the staining process, the design and preparation of the restoration, and the patient's individual factors such as antagonist, occlusion and habits [39]. It is important for dental professionals to carefully select and prepare the restoration properly to optimize its durability and longevity. Overall, with proper design, fabrication, and care, C + G restorations can provide good and excellent esthetic results.

The choice of the surface parameter Ra, or the arithmetical mean roughness, is justified in restorative dentistry due to its widespread acceptance and relevance in assessing roughness [11]. While parameters like Rz (maximum height of the profile) or Rt (total height variation) have their own merits and applications in specific contexts, Ra is commonly preferred in dentistry for providing an average measure of surface irregularities and is less affected by isolated peaks or valleys, making it a comprehensive evaluation of surface roughness [11, 12]. It is relatively easy to measure and calculate, making it accessible for routine clinical evaluations. Additionally, studies have shown correlations between higher Ra values and increased plaque retention, compromised surface integrity, and impaired esthetics. By prioritizing the reduction of Ra, clinicians can strive to provide restorations with smoother surfaces, enhancing patient comfort and improving esthetic outcomes [11, 12, 14, 24, 30].

As study's limitations, the absence of thermocycling tests would be necessary for investigating the mechanical behavior of the present zirconia in a more dynamic scenario [39, 40]. These tests involve subjecting a material to repeated thermos-cycles to simulate the stresses and strains that occur in the oral environment during oral functions [41]. Further studies could be developed including factors such as complex specimen, the amount of deformation and damage that occurs, and the material's fatigue strength with the evaluated finishing protocols. In addition, considering adhesively bonded restorations [42–44].

The thickness of the finishing layer plays a significant role in influencing the outcomes, as observed in the C + G group where the layer is thicker. This discrepancy likely impacted the results. In the context of Yttria-Stabilized Tetragonal Zirconia Polycrystal (YTZP), a prior study demonstrated a glaze layer with a thin and uniform surface, measuring between 24 and 28 *µ*m [45, 46]. In contrast, the same material in the C + G group was reported to have a thickness of 40.5 *μ*m in another study by Dal Piva et al. [4]. This variation in thickness adds complexity to the interpretation of the results and underscores the importance of considering these factors when analyzing the findings, suggesting that the positive outcome for C + G could have been promoted by the thicker specimen.

It is crucial to exercise caution when interpreting the results of this study, considering the in vitro nature of the setting and the need for further clinical trials to confirm the findings [43]. As limitation, no analysis was conducted to verify the standardization of the glaze layer, such as measuring sample thickness before and after application. The glaze layer application was standardized by a dental lab expert, performed by a single operator. Moreover, despite this study being carried out under tightly controlled laboratory conditions, its outcomes may not necessarily translate to real-world clinical scenarios [32].

## 5. Conclusion

The results of the study demonstrated that polished zirconia exhibited the lowest survival probability compared to glazed zirconia, indicating that glazing promotes higher long-term fatigue strength. Therefore, the application of either an extrinsic characterization followed by a glaze layer or a glaze layer as surface finishing protocols can enhance the survival probability of high-translucent zirconia, irrespective of the increase in surface roughness. These findings suggest that the additional steps of characterization and glazing contribute to the improved durability and longevity of zirconia restorations, outweighing the potential impact of increased surface roughness. Clinicians can therefore consider both surface finishing protocols to enhance the survival probability of high-translucent zirconia restorations while achieving satisfactory esthetic outcomes.

## Figures and Tables

**Table 1:** Mean fatigue strength (MPa) and cycles to fracture of the studied groups.

Groups	Mean fatigue strength (MPa)	CI
C	172.50^C^	159.43–185.56
C + G	192.75^B^	186.61–198.88
G	243.00^A^	222.36–263.63

*Note*: Different letters indicate statistically significant differences in the column. Means and 95% confidence intervals (CI).

**Table 2:** Survival probability (%) of experimental groups according to applied load and number of cycles.

Fracture load (N)/cycles	*C*	C + G	G
120/20,000	0.95	1	1
135/40,000	0.85	1	1
150/60,000	0.60	1	1
165/80,000	0.50	0.95	0.95
180/100,000	0.35	0.65	0.95
195/120,000	0.20	0.20	0.85
210/140,000	0.05	0.05	0.75
225/160,000	0	0	0.50
240/180,000	—	—	0.35
255/200,000	—		0.25
270/220,000	—	—	0.15
285/240,000	—	—	0.10
300/280,000	—	—	0.10
315/300,000	—	—	0.10
330/320,000	—	—	0.10
345/340,000	—	—	0.05
360/360,000	—	—	0.00

*Note*: Data show the survival probability, where 1 = 100% and 0 = 0%.

## Data Availability

Data supporting this research article are available from the first or last authors on request.
